# Evaluating large language models for clinical note processing: local fine-tuning and internal-external validation using electronic health records from South Asia

**DOI:** 10.1186/s12911-026-03366-8

**Published:** 2026-02-25

**Authors:** Seyed Alireza Hasheminasab, Faisal Jamil, Muhammad Usman Afzal, Ali Haider Khan, Sehrish Ilyas, Ali Noor, Awais Touseef, Salma Abbas, Hajira Nisar Cheema, Muhammad Usman Shabbir, Iqra Hameed, Maleeha Ayub, Hamayal Masood, Amina Jafar, Amir Mukhtar Khan, Muhammad Abid Nazir, Muhammad Asaad Jamil, Faisal Sultan, Sara Khalid

**Affiliations:** 1 Centre for Statistics in Medicine (CSM), Nuffield Department of Orthopaedics, Rheumatology and Musculoskeletal Sciences (NDORMS), University of Oxford, Oxford, UK; 2 Shaukat Khanum Memorial Cancer Hospital and Research Centre, Lahore, Punjab Pakistan

**Keywords:** Large language models, Clinical note processing, Electronic health records, Fine-tuning, External validation, Medical concept extraction, Medical question answering, Health equity, Global health

## Abstract

**Objective:**

Large Language Models (LLMs) hold the potential for clinical task-shifting by processing unstructured clinical text, enabling tasks such as clinical concept extraction and medical question answering from electronic health records. If implemented reliably, such approaches may benefit over-burdened healthcare systems, particularly in resource-limited settings and for traditionally overlooked populations, provided that local fine-tuning is supported by appropriate clinical and technical expertise. However, this powerful technology remains largely understudied in real-world contexts, particularly in the Global South. This study aims to assess whether openly available LLMs can be used reliably for processing medical notes in real-world settings in South Asia.

**Methods:**

We used publicly available LLMs to parse de-identified clinical notes from a large electronic health records (EHR) database in Pakistan, containing hospital records for 8.2 million patients. ChatGPT (GPT-3.5) as a general-purpose LLM, and GatorTron (base), BioMegatron, BioBert and ClinicalBERT as medical LLMs were evaluated when applied to these data, after fine-tuning them with (a) publicly available clinical datasets namely Informatics for Integrating Biology & the Bedside (I2B2) and National NLP Clinical Challenges (N2C2) for medical concept extraction (MCE) and emrQA for medical question answering (MQA), and (b) the local Pakistani de-identified EHR dataset, which includes inpatient Discharge Summaries (DS) and Subjective, Objective, Assessment, and Plan (SOAP) notes, as detailed in this paper. MCE models were applied to these clinical notes using both 3-label and 9-label formats, while MQA models were applied to medical questions. Internal and external validation performance was measured for (a) and (b) using F1 score, precision, recall, and accuracy for MCE and BLEU and ROUGE-L, which measure lexical and sequence similarity, for MQA.

**Results:**

When clinical LLMs were not fine-tuned on the local EHR dataset, their performance during external validation on local data was notably poorer compared to internal validation on the dataset used for fine-tuning, with reductions of at least 15% in F1 scores for MCE and 35% in ROUGE-L and BLEU scores for MQA tasks. This suggests potential bias and highlights the inability of the medical LLMs to reliably handle the data distribution of the local population without further fine-tuning and adaptation. This trend persisted across two distinct natural language processing tasks: concept extraction and question answering, spanning a spectrum of task complexities. However, fine-tuning the LLMs with local EHR data significantly improved model performance across both tasks, yielding a 7.5% to 15% increase in the F1 score for MCE and a 27% to 53% increase in ROUGE-L and BLEU scores for MQA. Notably, ChatGPT, as a general-purpose LLM, stood out as an exception, demonstrating superior performance across all measured metrics on the local dataset compared to the publicly available dataset, with improvements ranging from 3% to 17% on the local EHR dataset, even without fine-tuning on the local data.

**Conclusions:**

Publicly available LLMs, predominantly trained on data from high-income regions, were found to be unreliable when applied in a real-world clinical setting in Pakistan. Fine-tuning them with local EHR data and regional clinical contexts improved their reliability, demonstrating a feasible adaptation strategy that is substantially less resource-intensive than training large language models from scratch. Close collaboration between local clinical and technical experts to curate and leverage more representative, inclusive, and unbiased medical datasets, can play a crucial role in further ensuring reliability of LLMs for resource-limited, overburdened settings, to be used in ways that are safe, fair, and beneficial for all.

**Supplementary Information:**

The online version contains supplementary material available at 10.1186/s12911-026-03366-8.

## Introduction

### Background: large language models in clinical NLP

Large Language Models (LLMs) have emerged as powerful tools for processing unstructured clinical text, offering new opportunities to support clinical decision-making, task-shifting, and knowledge extraction from EHRs. By leveraging the power of transformer architectures trained on vast corpora of medical text, LLMs can extract, summarise, and contextualise clinical information at scale, enabling downstream applications such as clinical concept extraction, medical question answering, and summarisation of clinical notes. These applications may inform decision support for frontline healthcare workers, clinical trial selection, medical training, and medical data discovery [1–4]. If done reliably and rapidly, such use of LLMs can benefit over-burdened healthcare systems everywhere, but particularly in resource-limited settings such as South Asia, home to a quarter of the world’s population, and where rural and urban public health facilities are largely over-subscribed and under pressure.

Several medical-domain LLMs have been developed to address the linguistic and semantic characteristics of clinical text. Models such as ClinicalBERT, BioBERT, BioMegatron, and GatorTron are typically pretrained on biomedical literature and clinical corpora, including PubMed abstracts and intensive care records, to learn domain-specific representations [5–9]. Additional fine-tuning enables LLMs to perform specific tasks such as medical concept extraction, medical relation extraction, natural language inference, semantic textual similarity, medical event prediction, and medical question answering, while substantially reducing computational and data requirements compared to full pretraining [10]. 

EHRs, particularly free-text clinical notes, represent rich but underutilised repositories of information to aid patient care [11]. Key subjective information including family history, adverse drug events, and social, behavioural, and environmental determinants of health, all of which are commonly required in time-critical decision-making, is well-documented only within the full-text patient notes of EHRs rather than structured fields [12]. The lack of syntactic, structural, and semantic standardisation across EHR systems limits the reuse of this information for clinical care and research [13]. LLMs offer a potential mechanism to overcome these challenges by enabling scalable interpretation and extraction of information from unstructured clinical text [5]. Used in combination with structured data, this information can provide key contextual, socio-demographic and cultural nuances to improve health care, especially for traditionally marginalised communities with limited health access and representation in health data.

### Background: evaluation metrics and benchmarking in clinical NLP

Performance in clinical NLP tasks is commonly assessed using standard quantitative metrics. For medical concept extraction, precision, recall, and F1 score are widely used to evaluate token-level classification accuracy, particularly in benchmark datasets such as I2B2 and N2C2 [14, 15] These metrics quantify the trade-off between correctly identifying relevant clinical entities and avoiding false positives, and they form the basis of most reported comparisons between medical language models. For medical question answering tasks, evaluation typically relies on BLEU and ROUGE-L scores, which measure lexical overlap and sequence similarity between model-generated responses and reference answers [16, 17] These metrics are standard in datasets such as emrQA and are widely used to assess the quality of generated clinical responses [18]. 

In benchmark-based evaluations, medical LLMs fine-tuned and tested on matched datasets such as I2B2 and N2C2 typically achieve F1 scores in the range of approximately 0.85–0.95 for medical concept extraction, with similarly high BLEU and ROUGE-L scores reported for medical question answering tasks when training and test distributions are aligned [5–8]. These results have contributed to a perception of strong and stable model performance in the literature. However, such evaluations rarely assess robustness under external validation, where training and deployment data differ in clinical context, documentation style, language use, or population characteristics [19, 20].

### Background: dataset shift, bias, and generalisability

LLMs may hold promise to improve healthcare and reduce health disparities through their ability to process these data-rich sources and provide critical information to clinicians. The use of these models could hold significant benefits for traditionally overlooked groups, such as women, children, and socio-economically disadvantaged populations. However, these benefits are only achievable if the models are trained on diverse, inclusive datasets and utilize bias mitigation techniques to minimize the introduction and impact of biases.

A major limitation of existing evaluations is their reliance on homogeneous datasets derived largely from high-income, Global North healthcare systems. As a result, models trained and validated exclusively on these datasets may encode implicit biases related to documentation style, clinical practice, language use, and patient demographics. Ensuing models may therefore be under-representative of low-income geographies and populations, resulting in implicit assumptions about gender, race, and geography, socio-economic status, etc. Concealed biases within LLMs could cause performance degradation due to dataset shift, raising concerns about reliability, fairness, and safety with potentially severe repercussions on patient outcomes, and render them unsuitable for use with diverse, global populations [21–24]. 

Despite growing interest in the equitable deployment of LLMs in healthcare, there remains limited empirical evidence assessing how medical LLMs generalise across geographic and socio-cultural contexts. In particular, few studies have systematically evaluated model performance under external validation in real-world Global South clinical settings, or quantified the extent to which local fine-tuning can mitigate performance loss across multiple clinical NLP tasks.

### Study objectives and contributions

**Fig. 1:**
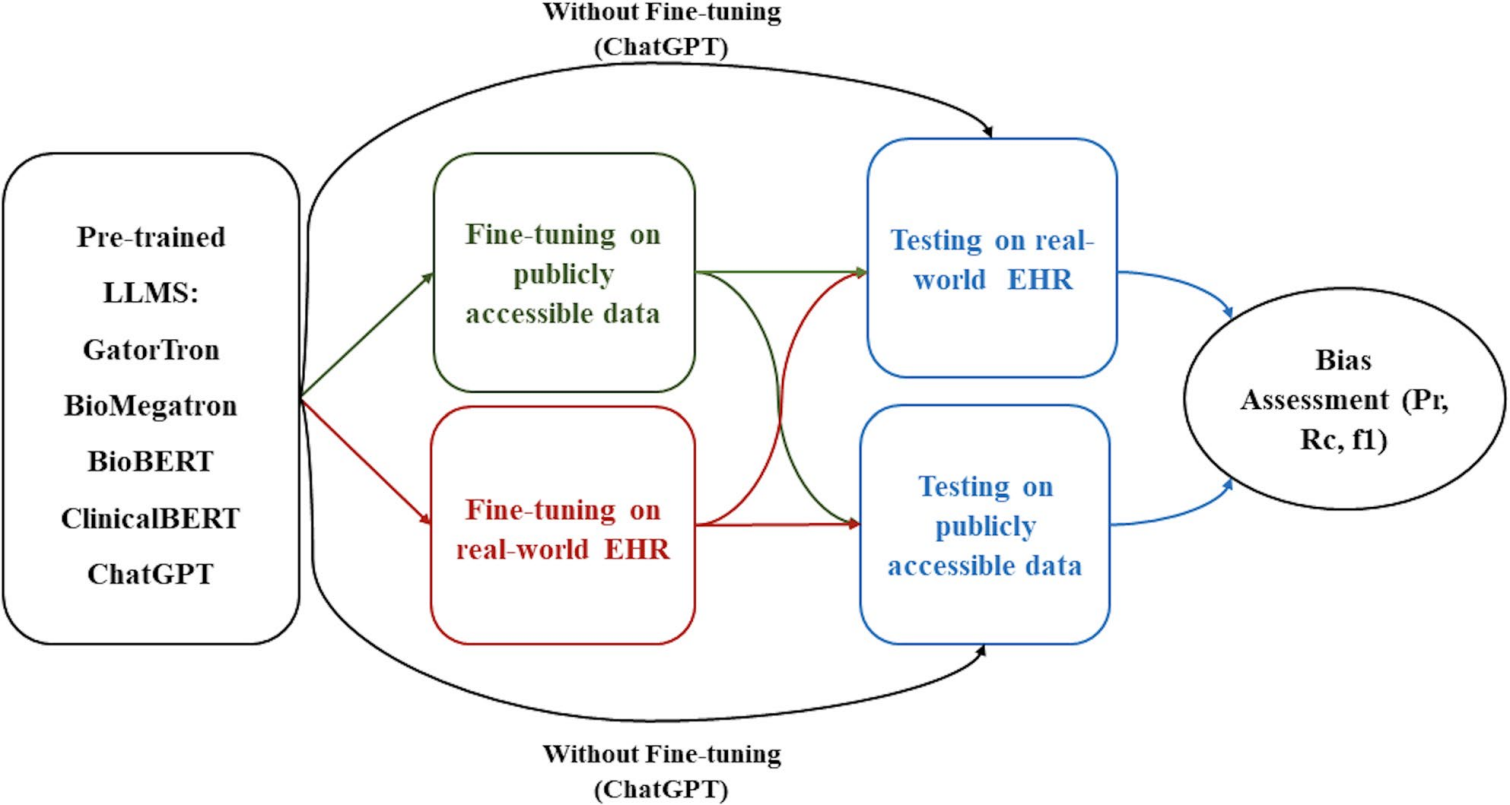
Study design for assessing bias of LLMs in real-world clinical settings. Internal and external validation was undertaken with and without fine-tuning on real-world data

In this paper, we studied the strengths and limitations of LLMs in a real-world, Global South clinical setting. We tested, and independently validated, publicly available LLMs on a large local EHR database in South Asia. To assess the reliability of these models, we compared performance with and without fine-tuning to the local dataset. We assessed both internal and external validation by testing the performance of models fine-tuned on the local EHR dataset on open datasets, and vice versa (Fig. 1). We evaluate two clinically relevant NLP tasks, medical concept extraction and medical question answering, to quantify the extent to which local fine-tuning improves robustness and generalisability under distribution shift. This approach is disease-agnostic and generalisable to other downstream uses of LLMs such as summarisation, inference and more. Key contributions of this study include a demonstration of the challenges and opportunities in the use of LLMs in real-world, resource-limited settings.

Despite rapid technical advances, clinical adoption of LLMs remains limited. Little is known about how these models perform in real-world settings, or whether they can be trusted to operate fairly, safely, and ethically across diverse clinical contexts [10, 25–30]. The lack of robust external validation has further hindered adoption and clinical trust, particularly in resource-limited healthcare systems. These gaps underscore the need for empirical evaluations of LLM reliability using real-world clinical data, such as those undertaken in this study. This work opens up avenues to further study the potential of LLMs to empower clinical decision-making and enable task-shifting which is beneficial for all.

## Methods

### Data source

The Shaukat Khanum Memorial Cancer Hospital and Research Centre (SKMCH&RC) (www.shaukatkhanum.org.pk) is a secondary and tertiary care hospital network spanning 70 cities in Pakistan. Its electronic health records database contains free-text notes and structured data for 8.2 million actively registered patients (51% women) [31]. It is linked with the Punjab Cancer Registry and contains anonymised, de-identified patient-level data on socio-demographics, laboratory results, clinical history, diagnoses, outcomes, prescriptions/dispensations, hospital in-patient procedures, and mortality from December 1994 to the present (1st June 2022). The SKMCH&RC dataset contains two types of free-text notes as follows.

#### Inpatient discharge summary notes (DS)

DS notes represent a comprehensive summary of a patient’s hospitalization, including diagnostic information, procedures performed, medications administered, and post-discharge instructions. Patient demographics, admission and discharge dates, primary consultants, and detailed information about the patient’s condition are documented under “Diagnosis During This Admission,” “Background Medical Problem(s),” and “Management During Admission” headings. These data can provide subjective information not necessarily captured in structured codes.

#### Subjective, objective, assessment, and plan notes (SOAP)

SOAP notes offer a structured approach to documenting patient information in 4 sections.

1. Subjective: Patient symptoms, history, and any information provided by the patient or caregiver.
2. Objective: Objective observations, laboratory results, and imaging data.
3. Assessment: Diagnosis, problem list, and a summary of the patient’s health status.
4. Plan: Detailed plans for treatment, medications, follow-up, and any other relevant actions.

SOAP notes provide a nuanced understanding of patient cases, ranging from diagnostic workups to treatment plans. Key entities in the dataset include patient demographics, medical history, diagnostic findings, treatment plans, and follow-up instructions.

### Labelling clinical notes

A team of six clinical experts including resident doctors labelled the DS and SOAP notes both for concept extraction and question answering tasks. Using a consensus approach, an answer with the highest level of agreement between the team was considered the “true” label, through majority voting. The labelled dataset was double-checked by the resident supervisory doctor. A token was considered to be the smallest unit of text that a given model can read such as a word, sub-word, or character.

For medical concept extraction, we used a standard named entity recognition (NER) approach, which aims to identify and label clinically meaningful phrases, such as diagnoses, medications, or procedures, within free-text clinical notes. The text was first divided into tokens (e.g. words or sub-words), and each token was assigned a label indicating whether it marked the beginning of a clinical concept, continued an existing concept, or lay outside any concept. Specifically, we used the Beginning-Inside-Outside (BIO) labelling scheme, where “B” denotes the first token of a clinical concept, “I” denotes subsequent tokens belonging to the same concept, and “O” denotes tokens not associated with any clinical concept. This representation allows models to learn both the identity and the boundaries of clinically relevant information within the target text.

### Medical LLMs

In addition to OpenAI’s ChatGPT as a trained general-purpose language model, 4 publicly available medical large language models (Table 1) designed for parsing medical notes were used, namely GatorTron (base), BioMegatron, ClinicalBERT, and BioBERT [5–8]. At the time of writing, these open-source models are available pre-trained on extensive medical datasets, allowing them to acquire a nuanced understanding of both medical terminology and English text structures. Domain-specific pretraining has been shown to significantly outperform mixed-domain approaches, particularly in biomedical NLP tasks [9]. 

**Table 1:** Pre-trained clinical LLMs used in the study with training datasets

Model Name	Model Size	Training Data
GatorTron base	354 M parameters	Clinical narratives from the University of Florida Health Integrated Data Repository (US), MIMIC III (US) Combining PubMed abstracts and full-text commercial-collectionWikipedia articles dump
BioMegatron	334 M parameters	Wikipedia CC-Stories Real news Open Web text PubMed abstract set Commercial Use Collection of the PubMed Central^®^ full-text corpus
ClinicalBERT	135 M parameters	MIMIC III (US)
BioBERT	107 M parameters	PubMed abstracts PubMed Central full-text articles
ChatGPT	not disclosed officially	Specific data sources are not publicly disclosed. A variety of sources, including publicly available web pages, books, and code repositories

### Fine-tuning LLMs

Each LLM is available pre-trained on large volumes of data (Table 1). In this study, we fine-tuned each pre-trained LLM for the task of parsing DS and SOAP notes through named entity recognition (NER), which involves identifying specific entities in medical records. Aside from ChatGPT, which was trained by OpenAI as a general-purpose LLM, each LLM was separately fine-tuned on (a) publicly accessible datasets I2B2 2010 (3 labels: treatment, test, problem), and N2C2 (9 labels: Duration, Frequency, Strength, Form, Route, Dosage, Reason, ADE, Drug), and emrQA (for question answering task), and (b) the SKMCH&RC dataset for each corresponding task. The emrQA dataset is derived from clinical notes originating from U.S. healthcare systems and represents a publicly available MQA benchmark developed in a high-income setting. Publicly accessible datasets were pre-segmented into training and testing sets. The SKMCH&RC dataset was randomly split into training (80%) and test (20%) sets. The detailed comparison of the datasets used for fine-tuning is presented in appendix 1.

### Model performance: internal and external validation

LLMs fine-tuned on I2B2 2010, N2C2, and emrQA training sets were internally validated on their respective test sets. They were then externally validated on the SKMCH&RC test set.

Similarly, LLMs fine-tuned on the SKMCH&RC train set were internally validated on the SKMCH&RC test set and externally validated on the I2B2 2010, N2C2, and emrQA test sets. ChatGPT was tested on the SKMCH&RC dataset without fine-tuning.

The detailed confusion matrices are presented as supplementary material to this paper.

### Evaluation of the models’ reliability

Model performance was measured using F1 score, precision, recall, and accuracy for the medical concept extraction task. For the medical question answering task, we used BLEU and ROUGE-L, which are standard evaluation metrics for text generation tasks. BLEU (Bilingual Evaluation Understudy) provides an estimate of lexical similarity, measuring the precision of n-gram overlap between generated answers and reference answers penalised for short answers, while ROUGE-L measures the longest common subsequence between generated and reference texts, capturing recall-oriented similarity [16, 17] These metrics are widely used in clinical question answering benchmarks, including emrQA, and enable consistent comparison across models, while acknowledging that they primarily assess surface-level textual similarity rather than deeper semantic equivalence [18]. Confusion matrices were produced to assess label-specific misclassification.

### Computational resources and execution time

All the presented open-source LLMs are accessible through the Hugging Face website at no cost, except for ChatGPT, which requires a paid subscription. To conduct fine-tuning and evaluate model performance, we utilized a Google Colaboratory account with a paid subscription, equipped with an NVIDIA A100 GPU. Fine-tuning and evaluation were performed separately for each model and task using identical computational settings where applicable. Execution times reported in Table 2 represent approximate wall-clock times and are intended to provide an order-of-magnitude estimate rather than precise benchmarking, as runtimes may vary depending on implementation details and system load.

**Table 2:** Approximate computational resources and execution times for fine-tuning and evaluation of LLMs

Task	Model type	Fine-tuning dataset	Compute environment	Approx. fine-tuning time	Approx. evaluation time
Medical Concept Extraction (3-label)	Open-source LLMs	Public (I2B2)	A100	~ 2.1 h	~ 10 min
Medical Concept Extraction (9-label)	Open-source LLMs	Public (N2C2)	A100	~ 4 h	~ 15 min
Medical Concept Extraction (local 3-label)	Open-source LLMs	SKMCH&RC	A100	~ 1.3 h	~ 10 min
Medical Concept Extraction (9-label)	Open-source LLMs	SKMCH&RC	A100	~ 1.5 h	~ 10 min
Medical Question Answering	Open-source LLMs	emrQA	A100	~ 12 h	~ 1 h
Medical Question Answering (local)	Open-source LLMs	SKMCH&RC	A100	~ 3 h	~ 30 min
Inference (ChatGPT)	ChatGPT (API)	SKMCH&RC	Cloud API	Not applicable	~ 2–3 s per query

Execution times are approximate and reflect single-run experiments on a single NVIDIA A100 GPU. Fine-tuning times for medical question answering tasks are longer than those for concept extraction due to the sequence-generation objective and longer input–output lengths. Fine-tuning times are substantially lower than those required for training large language models from scratch, which typically require distributed multi-GPU or multi-node infrastructure.

## Results

A total of 200 free-text notes, including 50 DS and 150 SOAP notes were randomly extracted from the SKMCH&RC dataset for a two-year period from 01-Jan-2020 to 21-Nov-2021 and labelled by clinical experts. One note per patient was extracted; the notes represented a patient population including 46% men and 54% women; 89% were adults (defined as aged 19–87 years).

This included 284,445 expert-labelled tokens in BIO format, including 140,841 tokens representing the 3 classes and 143,604 tokens representing the 9 classes. In comparison, the public datasets contained a total number of 2,485,556 BIO tokens, including 1,314,036 tokens representing the 3 classes and 1,171,520 tokens representing the 9 classes. Table 3 displays fine-tuning and internal/external testing dataset statistics in concept extraction task.

**Table 3:** Overview of datasets employed for fine-tuning and internal/external validation in concept extraction task

Name	Publicly accessible benchmark datasets (US)		Real-world EHR database (Pakistan)	
	I2B2-2010	N2C2-2018	SKMCH&RC	SKMCH&RC
N labels	3 Labels	9 Labels	3 Labels	9 Labels
sample sizes	170 Train files,	204 Train files,	200 files	200 files
	256 Test files	204 Test files		
train/validation splits	90/10	80/20	Train (60%), validation (10%), and test (30%)	Train (60%), validation (10%), and test (30%)
	Train: 152,	Train: 163	Train: 120	Train: 120
	Validation: 18	Validation: 41	Validation: 20	Validation: 20
	Test: 256	Test: 204	Test: 60	Test: 60
Notes Structure	Original text from I2B2_2010 files	Original text from N2C2_2018 files	150 SOAP notes and 50 Discharge Summaries	150 SOAP notes and 50 Discharge Summaries
Labels Number of resulting BIO labels	[‘treatment’, ‘test’, ‘problem’] **N of BIO labels = 7**	[‘Duration’, ‘Frequency’, ‘Strength’, ‘Form’, ‘Route’, ‘Dosage’, ‘Reason’, ‘ADE’, ‘Drug’] **N of BIO labels = 19**	[‘treatment’, ‘test’, ‘problem’] **N of BIO labels = 7**	[‘Duration’, ‘Frequency’, ‘Strength’, ‘Form’, ‘Route’, ‘Dosage’, ‘Reason’, ‘ADE’, ‘Drug’] **N of BIO labels = 19**

### Model performance

The performance of the models for internal and external validation of the concept extraction and question answering tasks is summarized in Tables 4 and 5, respectively.

**Table 4:** Concept extraction model performance for internal and external validation of LLMs fine-tuned on publicly accessible and SKMCH&RC datasets. In this table, each row indicates whether fine-tuning and testing were conducted on public or SKMCH&RC datasets. By referencing the number of labels in each column, the dataset used can be inferred. For instance, the combination of “public” and “3-label” implies the utilization of the public dataset with a 3-label or I2B2 dataset

Model Name	GatorTron base	BioMegatron	ClinicalBERT	BioBERT	ChatGPT	GatorTron base	BioMegatron	ClinicalBERT	BioBERT	ChatGPT
Label Format	3-Label					9-Label				
Precision Finetuned on Public Tested on Public	**0.8303** **[**0.8256, 0.8368**]**	**0.8575** **[**0.8509, 0.8619**]**	**0.8612** **[**0.8567, 0.8651**]**	**0.8641** **[**0.8584, 0.8704**]**	**0.8114**	**0.9555** **[**0.9516, 0.9599**]**	**0.946** **[**0.9411, 0.9499**]**	**0.9316** **[**0.9254, 0.9376**]**	**0.9476** **[**0.9436, 0.9523**]**	**0.9124**
Recall Finetuned on Public Tested on Public	**0.8448** **[**0.8305, 0.8579**]**	**0.8736** **[**0.8617, 0.8826**]**	**0.8791** **[**0.8679, 0.888**]**	**0.869** **[**0.8578, 0.8788**]**	**0.76**	**0.9625** **[**0.9591, 0.9668**]**	**0.956** **[**0.9502, 0.9605**]**	**0.9462** **[**0.9419, 0.9512**]**	**0.9678** **[**0.9653, 0.9714**]**	**0.6978**
F1 Finetuned on Public Tested on Public	**0.8375** **[**0.8288, 0.8447**]**	**0.8655** **[**0.8569, 0.872**]**	**0.8701** **[**0.8623, 0.8746**]**	**0.8665** **[**0.8581, 0.8746**]**	**0.7667**	**0.959** **[**0.9556, 0.9628**]**	**0.951** **[**0.9461, 0.9546**]**	**0.9388** **[**0.9339, 0.9441**]**	**0.9576** **[**0.9548, 0.9615**]**	**0.7496**
Accuracy Finetuned on Public Tested on Public	**0.9584** **[**0.9564, 0.9607**]**	**0.9507** **[**0.9477, 0.9531**]**	**0.948** **[**0.9449, 0.9498**]**	**0.9463** **[**0.9429, 0.9492**]**	**0.76**	**0.9934** **[**0.993, 0.9938**]**	**0.9899** **[**0.9892, 0.9904**]**	**0.9934** **[**0.9859, 0.9875**]**	**0.9903** **[**0.9895, 0.9908**]**	**0.6978**
Precision Finetuned on Public Tested on SKMCH&RC	**0.5278** **[**0.5142, 0.5495**]**	**0.6575** **[**0.6323, 0.6746**]**	**0.6307** **[**0.6051, 0.646**]**	**0.6442** **[**0.6214, 0.6594**]**	**0.9214**	**0.7468** **[**0.728, 0.7667**]**	**0.7368** **[**0.7187, 0.7542**]**	**0.7468** **[**0.728, 0.7667**]**	**0.74** **[**0.7206, 0.7616**]**	**0.9156**
Recall Finetuned on Public Tested on SKMCH&RC	**0.644** **[**0.6195, 0.6726**]**	**0.7736** **[**0.7538, 0.7906**]**	**0.7522** **[**0.7321, 0.7664**]**	**0.7603** **[**0.7413, 0.7745**]**	**0.9118**	**0.8214** **[**0.81, 0.832**]**	**0.8278** **[**0.8181, 0.8365**]**	**0.8214** **[**0.81, 0.832**]**	**0.7941** **[**0.7822, 0.8093**]**	**0.8716**
F1 Finetuned on Public Tested on SKMCH&RC	**0.5801** **[**0.5629, 0.6019**]**	**0.7108** **[**0.6923, 0.728**]**	**0.6861** **[**0.6642, 0.6999**]**	**0.6974** **[**0.6808, 0.7103**]**	**0.9097**	**0.7823** **[**0.7729, 0.7961**]**	**0.7797** **[**0.7691, 0.7913**]**	**0.7823** **[**0.7729, 0.7961**]**	**0.766** **[**0.7545, 0.7847**]**	**0.8853**
Accuracy Finetuned on Public Tested on SKMCH&RC	**0.8906** **[**0.8833, 0.8973**]**	**0.8705** **[**0.8615, 0.8787**]**	**0.8498** **[**0.8406, 0.8573**]**	**0.8502** **[**0.8428, 0.8582**]**	**0.9118**	**0.9556** **[**0.9511, 0.9597**]**	**0.9446** **[**0.9391, 0.9497**]**	**0.9556** **[**0.9511, 0.9597**]**	**0.9229** **[**0.915, 0.9303**]**	**0.8716**
Precision Finetuned on SKMCH&RC Tested on SKMCH&RC	**0.7826** **[**0.7608, 0.8027**]**	**0.8328** **[**0.8175, 0.8529**]**	**0.8098** **[**0.7906, 0.8303**]**	**0.8243** **[**0.8016, 0.8424**]**		**0.8449** **[**0.8303, 0.8604**]**	**0.9115** **[**0.8967, 0.9225**]**	**0.8987** **[**0.8844, 0.9089**]**	**0.9093** **[**0.8947, 0.9184**]**	
Recall Finetuned on SKMCH&RC Tested on SKMCH&RC	**0.7812** **[**0.7639, 0.8007**]**	**0.8341** **[**0.8208, 0.8516**]**	**0.8188** **[**0.7994, 0.8388**]**	**0.8395** **[**0.8252, 0.8543**]**		**0.8704** **[**0.8556, 0.8846**]**	**0.9178** **[**0.9048, 0.9314**]**	**0.9076** **[**0.8981, 0.9214**]**	**0.9131** **[**0.8967, 0.9275**]**	
F1 Finetuned on SKMCH&RC Tested on SKMCH&RC	**0.7819** **[**0.7659, 0.8017**]**	**0.8334** **[**0.8204, 0.8521**]**	**0.8142** **[**0.7993, 0.8343**]**	**0.8318** **[**0.8136, 0.8468**]**		**0.8575** **[**0.8449, 0.8717**]**	**0.9146** **[**0.9015, 0.9263**]**	**0.9031** **[**0.8912, 0.914**]**	**0.9112** **[**0.8967, 0.9222**]**	
Accuracy Finetuned on SKMCH&RC Tested on SKMCH&RC	**0.9531** **[**0.9502, 0.9571**]**	**0.9399** **[**0.935, 0.9456**]**	**0.934** **[**0.9281, 0.9407**]**	**0.9382** **[**0.9319, 0.9431**]**		**0.9733** **[**0.9697, 0.9769**]**	**0.9761** **[**0.9733, 0.9792**]**	**0.9744** **[**0.9720, 0.9774**]**	**0.9737** **[**0.9700, 0.977**]**	
Precision Finetuned on SKMCH&RC Tested on Public	**0.4398** **[**0.4315, 0.4523**]**	**0.6317** **[**0.6217, 0.6429**]**	**0.6525** **[**0.6432, 0.6632**]**	**0.6274** **[**0.6172, 0.6384**]**		**0.6757** **[**0.6654, 0.686**]**	**0.7317** **[**0.7244, 0.7399**]**	**0.712** **[**0.703, 0.7229**]**	**0.7302** **[**0.724, 0.7378**]**	
Recall Finetuned on SKMCH&RC Tested on Public	**0.4068** **[**0.3963, 0.4194**]**	**0.5321** **[**0.5191, 0.5459**]**	**0.5948** **[**0.5811, 0.6094**]**	**0.5623** **[**0.5451, 0.5773**]**		**0.6291** **[**0.6166, 0.6463**]**	**0.6417** **[**0.6323, 0.6492**]**	**0.7112** **[**0.7002, 0.7253**]**	**0.6335** **[**0.6227, 0.646**]**	
F1 Finetuned on SKMCH&RC Tested on Public	**0.4226** **[**0.4146, 0.4337**]**	**0.5776** **[**0.5671, 0.5879**]**	**0.6223** **[**0.6121, 0.6336**]**	**0.593** **[**0.5799, 0.6039**]**		**0.6515** **[**0.643, 0.6625**]**	**0.6837** **[**0.6774, 0.6899**]**	**0.7116** **[**0.7043, 0.7204**]**	**0.6784** **[**0.6722, 0.6871**]**	
Accuracy Finetuned on SKMCH&RC Tested on Public	**0.8199** **[**0.8161, 0.8243**]**	**0.8269** **[**0.8209, 0.832**]**	**0.8379** **[**0.8315, 0.8421**]**	**0.8363** **[**0.83, 0.842**]**		**0.9524** **[**0.9503, 0.9541**]**	**0.9363** **[**0.9337, 0.9386**]**	**0.9488** **[**0.947, 0.9505**]**	**0.9343** **[**0.9322, 0.9364**]**	

**Table 5:** Question answering model performance for internal and external validation of LLMs fine-tuned on publicly accessible and SKMCH&RC datasets. In this table, each row indicates whether fine-tuning and testing were conducted on public or SKMCH&RC datasets

Model Name	GatorTron base	BioMegatron	ClinicalBERT	BioBERT	ChatGPT
BLEU Finetuned on Public Tested on Public	**0.9089** **[**0.9047, 0.9135**]**	**0.9081** **[**0.9038, 0.9133**]**	**0.8853** **[**0.8808, 0.8892**]**	**0.9056** **[**0.9000, 0.9099**]**	**0.5651** **[**0.5526, 0.578**]**
ROUGE-L Finetuned on Public Tested on Public	**0.9532** **[**0.9486, 0.9573**]**	**0.9534** **[**0.9491, 0.9589**]**	**0.9336** **[**0.9278, 0.9386**]**	**0.9502** **[**0.9444, 0.9559**]**	**0.6264** **[**0.6134, 0.6403**]**
BLEU Finetuned on Public Tested on SKMCH&RC	**0.4630** **[**0.4552, 0.4711**]**	**0.5127** **[**0.5063, 0.5208**]**	**0.201** **[**0.1951, 0.2083**]**	**0.2836** **[**0.2768, 0.2896**]**	**0.6621** **[**0.6555, 0.6699**]**
ROUGE-L Finetuned on Public Tested on SKMCH&RC	**0.5587** **[**0.5496, 0.5659**]**	**0.6037** **[**0.5960, 0.6098**]**	**0.2909** **[**0.2847, 0.299**]**	**0.382** **[**0.3741, 0.3875**]**	**0.742** **[**0.735, 0.7495**]**
BLEU Finetuned on SKMCH&RC Tested on SKMCH&RC	**0.8475** **[**0.8358, 0.8591**]**	**0.8222** **[**0.8077, 0.8367**]**	**0.7311** **[**0.7164, 0.7495**]**	**0.778** **[**0.7606, 0.7948**]**	
ROUGE-L Finetuned on SKMCH&RC Tested on SKMCH&RC	**0.8966** **[**0.8840, 0.9077**]**	**0.8766** **[**0.8658, 0.8864**]**	**0.7911** **[**0.7768, 0.8125**]**	**0.8408** **[**0.8269, 0.8553**]**	
BLEU Finetuned on SKMCH&RC Tested on Public	**0.4671** **[**0.4601, 0.4736**]**	**0.483** **[**0.4739, 0.4918**]**	**0.2888** **[**0.2812, 0.2987**]**	**0.3562** **[**0.3482, 0.3636**]**	
ROUGE-L Finetuned on SKMCH&RC Tested on Public	**0.5584** **[**0.5502, 0.5652**]**	**0.5666** **[**0.5587, 0.5754**]**	**0.3538** **[**0.3451, 0.3646**]**	**0.4254** **[**0.4161, 0.4329**]**	

**Fig. 2:**
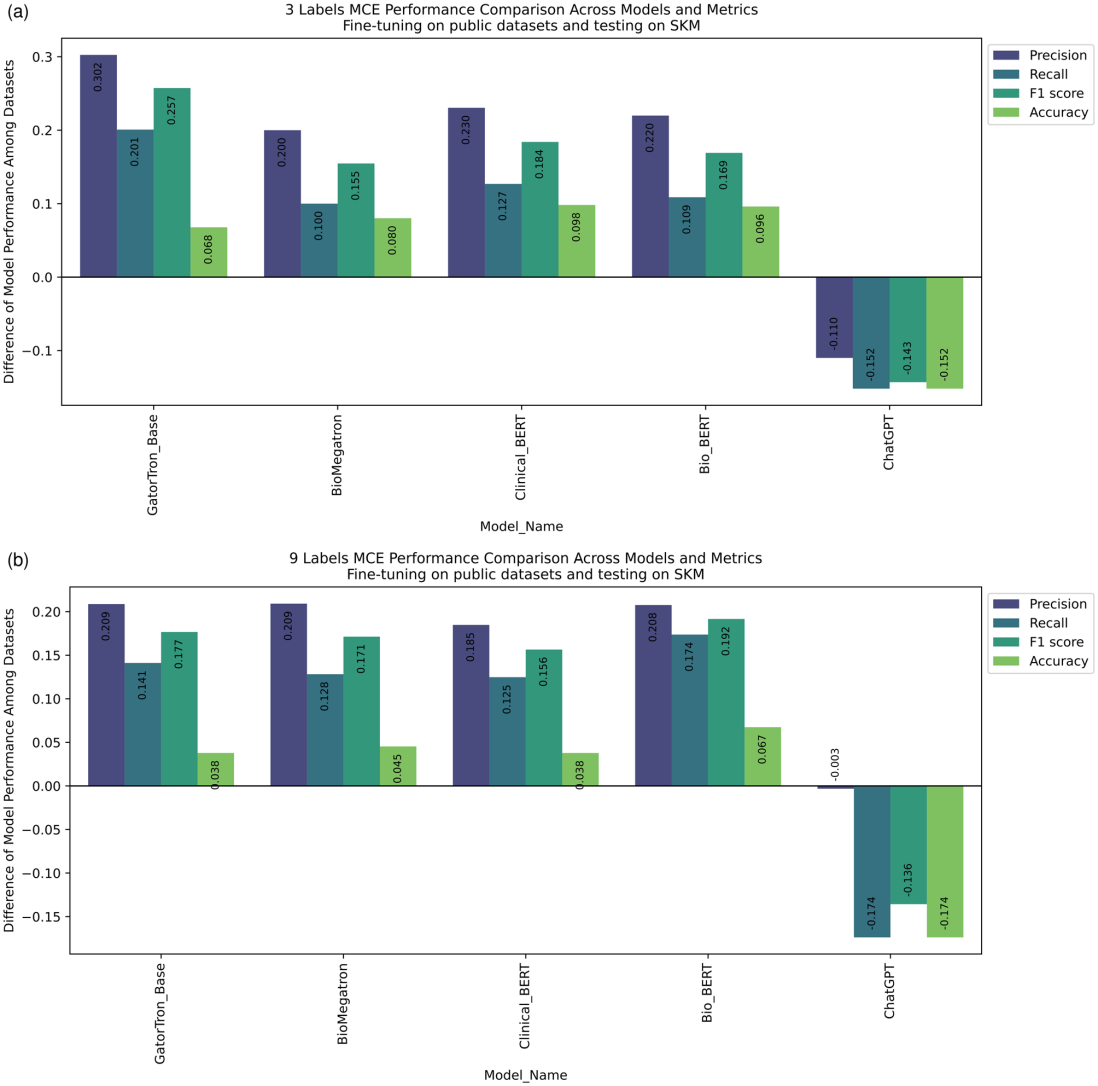
Difference between concept extraction performance on public and SKMCH&RC data compared between models. **a**) compares 3-label performance, **b**) compares 9-label performance

**Fig. 3:**
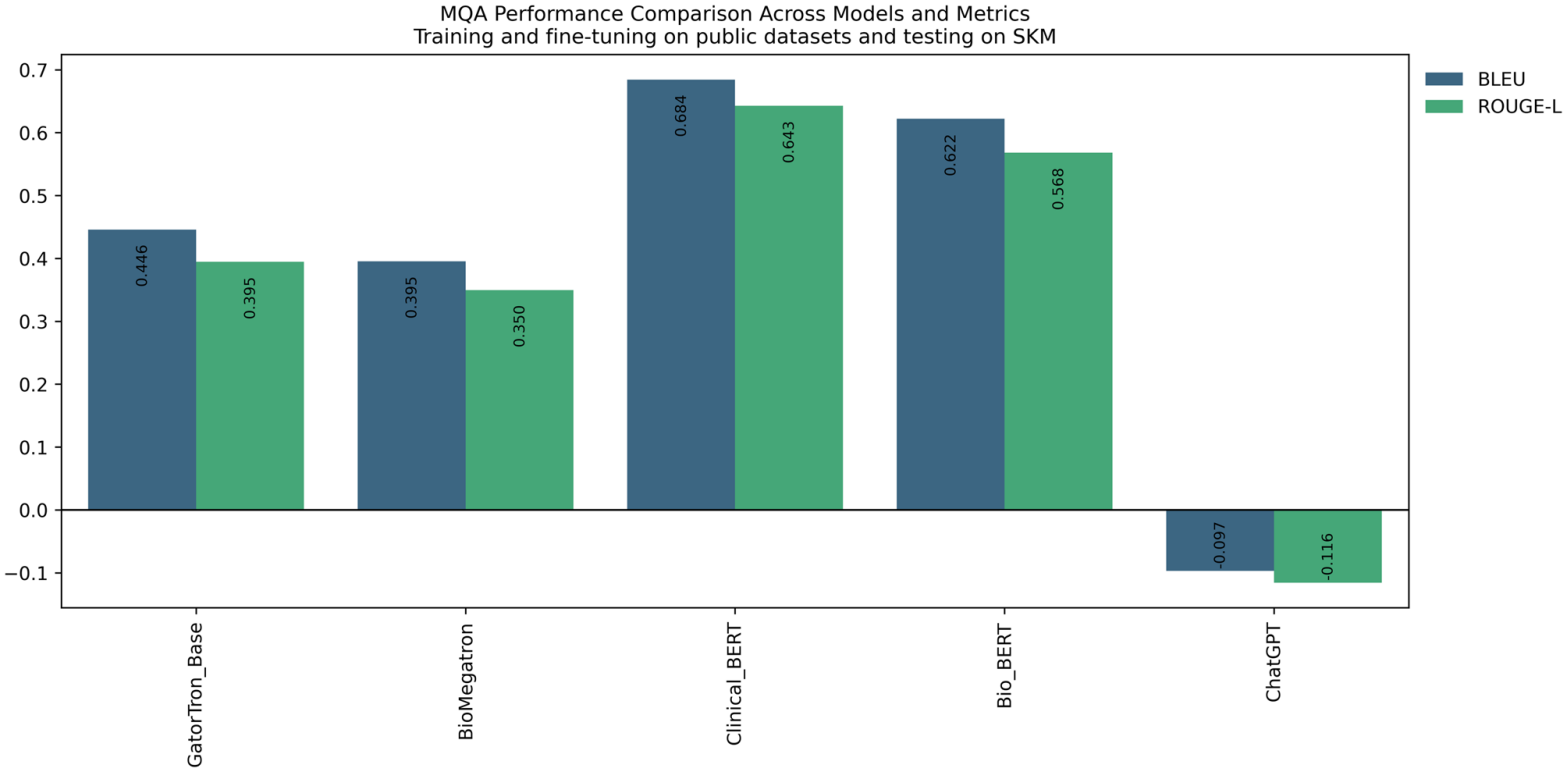
Difference between question answering performance on public and SKMCH&RC data compared between models

In general, for open-source LLMs, including GatorTron, BioMegatron, ClinicalBERT, and BioBERT, when we fine-tuned the models on publicly accessible data for the purpose of our NLP tasks, their performance significantly reduced when tested on SKMCH&RC, and vice versa in both NLP tasks. This observation indicates performance incompatibility in these models across different datasets, likely stemming from inherent biases in the data sources on which these models were trained. Interestingly, ChatGPT, whose source training datasets are not fully disclosed, exhibited superior performance on SKMCH&RC compared to publicly accessible datasets (Fig. 2a and b). This pattern persisted across other performance metrics such as accuracy, precision, and recall for concept extraction and BLEU and ROUGE metrics for question answering task (Fig. 3).

In general, LLMs fine-tuned on the SKMCH&RC training dataset resulted in the highest accuracy, precision, recall, and F1 score when tested on the SKMCH&RC test set. Specifically, the highest and lowest accuracy of 0.9531 (0.9502, 0.9571) and 0.934 (0.9281, 0.9407) belongs to GatorTron and ClinicalBERT respectively and the highest and lowest F1 score of 0.8334 (0.8204, 0.8521) and 0.7819 (0.7659, 0.8017) belongs to BioMegaTron and GatorTron in the dataset with 3-labels (I2B2). For the dataset with 9 labels (N2C2), BioMegaTron had the best performance with accuracy and F1 score of 0.9761 (0.9733, 0.9792), 0.9146 (0.9015, 0.9263) respectively, and GatorTron had the worst performance with 0.9733 (0.9697, 0.9769) and 0.8575 (0.8449, 0.8717).

For question answering task, GatorTron performs the best with BLEU and ROUGE-L scores of 0.8475 (0.8358, 0.8591) and 0.8966 (0.884, 0.9077) and ClinicalBERT was the worst performing with 0.7311 (0.7164, 0.7495) and 0.7911 (0.7768, 0.8125). ChatGPT produced BLEU of 0.6621 (0.6555, 0.6699) and ROUGE-L of 0.742 (0.735, 0.7495).

Models fine-tuned on public datasets I2B2 and N2C2 resulted in the highest accuracy, precision, recall, and F1 score when internally validated on their respective test sets. In the I2B2 dataset, GatorTron demonstrated superior performance in terms of accuracy, achieving a score of 0.9584 (0.9564, 0.9607). ClinicalBERT outperformed other models in recall and F1 score, attaining scores of 0.8791 (0.8679, 0.888) and 0.8701 (0.8623, 0.8746), respectively. BioBERT exhibited the highest precision, recording a value of 0.8641 (0.8584, 0.8704). Conversely, ChatGPT displayed the lowest performance across all metrics, yielding scores of 0.76 for accuracy, 0.8114 for precision, 0.7667 for F1 score, and 0.76 for recall. For the N2C2 dataset, GatorTron emerged as the top performer in terms of accuracy, precision, F1 score with 0.9934 (0.993, 0.9938), 0.9555 (0.9516, 0.9599), and 0.959 (0.9556, 0.9628) respectively and BioBERT had the best Recall of 0.9678 (0.9653, 0.9714). In contrast, ChatGPT exhibited the least favourable performance on this dataset, achieving scores of 0.6978 for accuracy, 0.9124 for precision, 0.7496 for F1 score, and 0.6978 for recall.

Highest performing models for question answering in open dataset was GatorTron with the highest BLEU of 0.9089 (0.9047, 0.9135) and BioMegaTron with the highest ROUGE-L of 0.9534 (0.9491, 0.9589) and ClinicalBERT had the worst BLEU and ROUGE-L of 0.8853 (0.8808, 0.8892), and 0.9336 (0.9278, 0.9386) respectively. ChatGPT was evaluated with BLEU of 0.5651 (0.5526, 0.578) and ROUGE-L of 0.6264 (0.6134, 0.6403).

When models fine-tuned on public datasets I2B2 and N2C2 were evaluated on the SKMCH&RC test set with three labels, a significant decline in performance was observed compared to models fine-tuned specifically on SKMCH&RC, as illustrated in Fig. 2a. This decline in performance persisted when evaluating the SKMCH&RC test set with nine labels (Fig. 2b). A similar pattern was evident in the question answering task (Fig. 3).

**Fig. 4:**
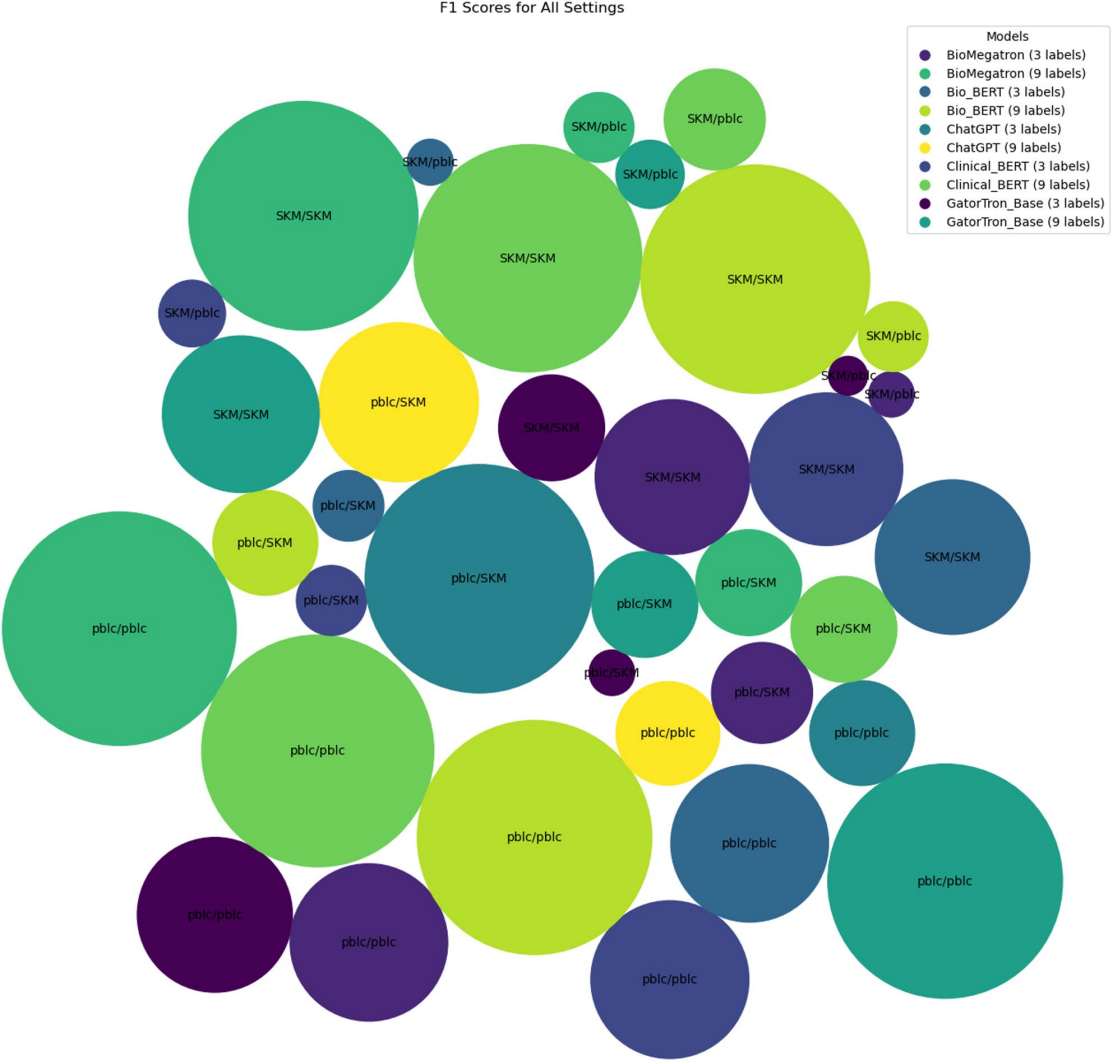
Bubble chart showing F1 scores for various LLMs across different train/test datasets. Bubble size indicates performance, with colours representing different models

Figure 4 presents the F1 scores of Language Models (LLMs) under different settings. Each LLM is depicted with a distinct colour, and the size of each circle corresponds to the F1 score of the models. The labels in the circles are divided by a “/“, where the text before the “/“ indicates the dataset used for fine-tuning, and the text after the “/“ signifies the datasets used for testing. Upon examination of the figure, a notable trend emerges: for each model designed to accommodate any number of labels, circles labelled with the same dataset for both fine-tuning and testing exhibit larger relative radii. This observed difference in radii can serve as an indicator of the dissimilarity in distributions between the two datasets, providing valuable insights into the impact of dataset variations on model performance.

**Fig. 5:**
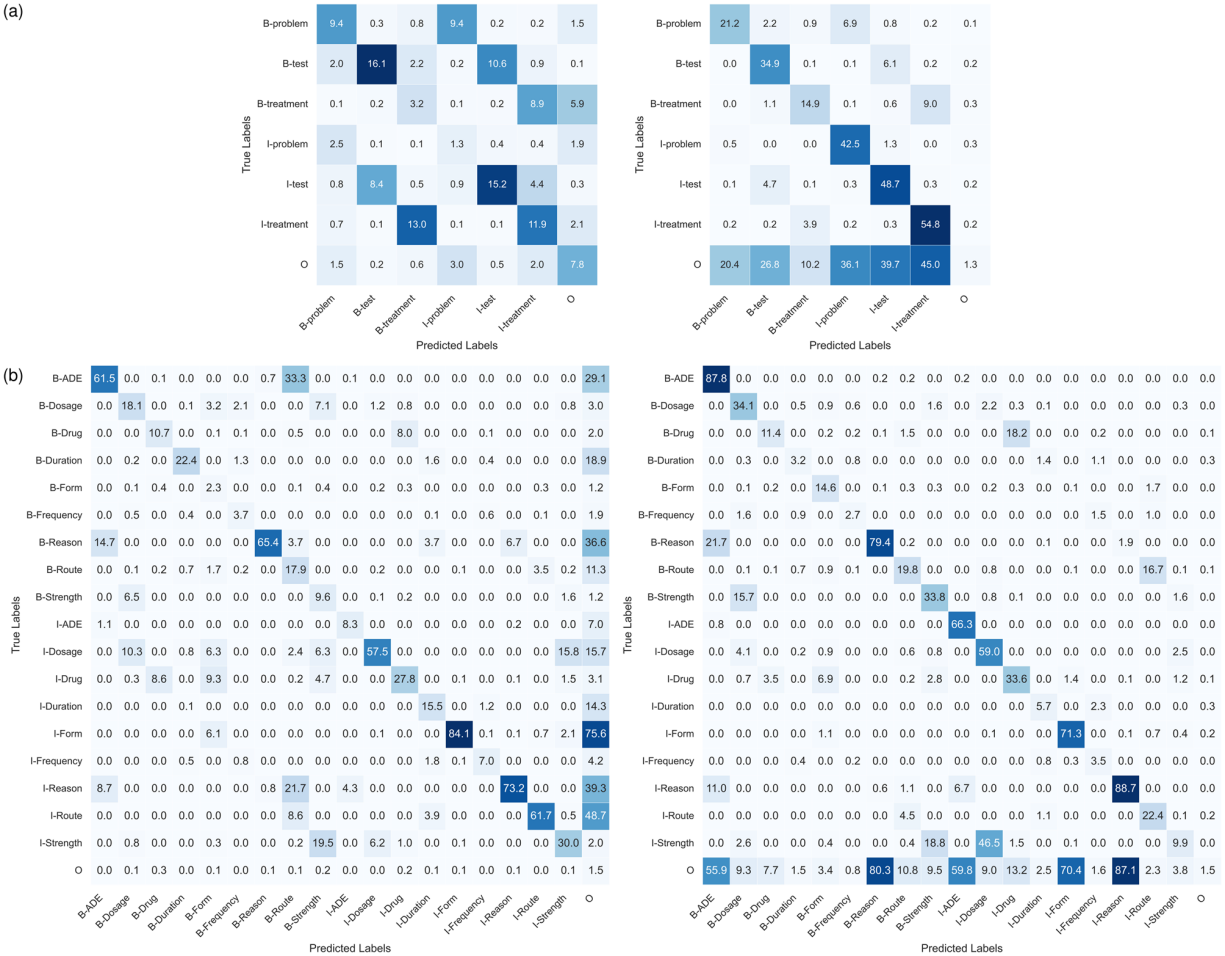
Absolute differences in normalized confusion matrices between true and predicted labels. The left tables show normalized to true labels, and the right tables show normalized to predicted labels; **a**) shows the 3-label I2B2 dataset difference to SKMCH&RC; **b**) shows the 9-label classification scheme (N2C2) highlighting label-specific variations in misclassification patterns across datasets

Figure 5 shows the label-specific misclassification performance for GatorTron, which had the largest drop in performance in internal (tested on I2B2) vs. external validation (tested on SKMCH&RC). The left of Fig. 5a showcases the absolute difference in model performances normalized by true labels, while the right highlights the differences between normalized confusion matrices based on predicted labels. Examining the diagonal elements of the matrices suggests differences in misclassification of all labels in the two testing datasets, with smaller differences observed in “B-Treatments.” In the 9-label dataset (Fig. 5b), the smallest differences were in the classification of the “B-Drug”, “B-Form”, “B-Frequency”, “I-Duration”, and “I-Frequency” labels. A similar pattern was observed for the other LLMs.

**Fig. 6:**
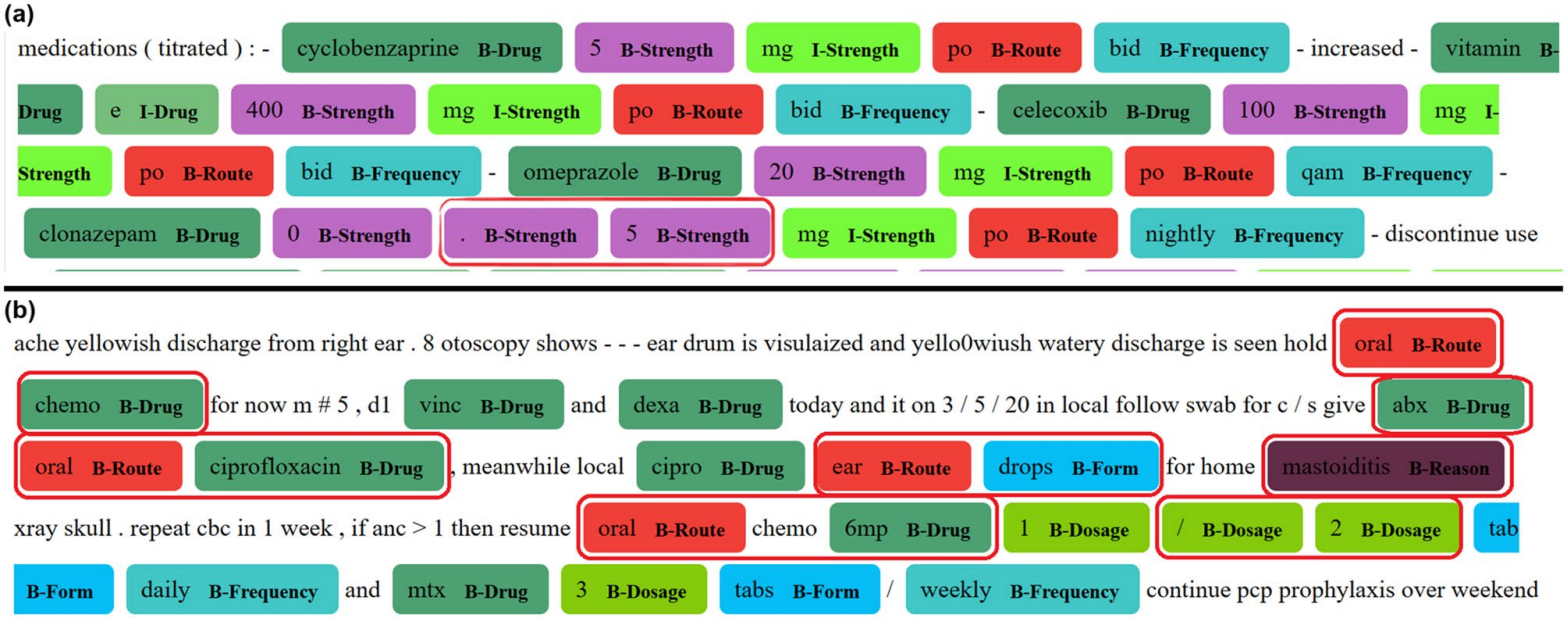
LLM labelling of 2 different clinical notes examples, demonstrating where the model performs well (**a**) and performs badly (**b**). Red boxes indicate misclassified labels; both examples were labelled by the GatorTron LLM using the 9-label classifications

Figure 6 displays two excerpts from sample notes within the SKMCH&RC dataset, each containing 9 labels (19 BIO labels), with true labels annotated by clinical experts and estimated labels generated by the GatorTron LLM. Figure 6a represents a snippet of text for which the LLM performed well in correctly predicting labels for the tokens, whereas Fig. 6b illustrates a case where the LLM performed poorly.

## Discussion

This section is structured to first summarise the principal findings, then interpret model behaviour and fine-tuning strategies, and finally reflect on practical implications and limitations for real-world deployment.

### Principal findings

In this study, we tested openly available medical LLMs in a real-world clinical setting in South Asia. To our knowledge, this is one of the few studies investigating the use of LLM technology in the global South, and the first study from Pakistan. All LLMs used in this work are publicly available (except for ChatGPT, which requires a paid subscription), rendering this approach reproducible in other settings without access and cost constraints. Models were tested on two forms of clinical notes (DS and SOAP notes) for the task of parsing clinical notes, making the approach disease-agnostic and generalisable. We evaluated model performance under both internal and external validation scenarios, with and without fine-tuning to a local EHR dataset. We further tested how well LLMs fine-tuned on the local dataset perform when tested on open-source medical datasets.

Across two distinct natural language processing tasks, medical concept extraction and medical question answering, we observed performance degradation when models fine-tuned on public datasets were applied to the local EHR data, and vice versa. In contrast, the same models, when fine-tuned on the local EHR database, performed well when tested on the local EHR database, albeit poorly on open datasets. These findings highlight the sensitivity of LLM performance to data distribution and underscore the limitations of relying solely on benchmark-based evaluations to assess real-world reliability.

Interestingly, ChatGPT, whose source training data are not fully disclosed (at the time of writing), was the exception to this trend, exhibiting better performance in terms of accuracy, F1 score, precision, recall, BLEU, ROUGE-L when tested on SKMCH&RC compared to publicly accessible datasets.

### Why local fine-tuning matters

Our findings suggest that off-the-shelf LLMs can be unreliable when directly applied to real-world clinical settings, as they tend to reflect biases inherent in the non-inclusive data sources on which they were trained. This is unsurprising given that the LLMs used in the study were trained on open-source datasets that are not representative of data from the real-world clinical setting we investigated.

For the models to be applicable in a localised setting, the training data must be representative of these populations, but local data used in isolation is often sparse and ungeneralisable for training an LLM entirely. Fine-tuning offers a viable approach to improving model reliability while saving time and resources compared to training an LLM from scratch, a process that is often prohibitively time- and resource-intensive, requiring expensive computing infrastructure that remains inaccessible to healthcare providers in resource-limited settings.

Importantly, our results also showed it is possible to reduce such data-driven bias by fine-tuning on locally representative data under expert supervision. Fine-tuning consistently improved model performance when applied within the same clinical context, supporting a pragmatic and resource-efficient alternative to training large language models from scratch. This is particularly relevant in resource-limited settings, where full model pretraining is often infeasible due to prohibitive computational and financial costs.

### Why ChatGPT behaved differently from other LLMs

An important observation in this study was that ChatGPT demonstrated comparatively strong performance on the local EHR dataset despite not being explicitly fine-tuned on local data, in contrast to the open-source medical LLMs evaluated. Although the proprietary nature of ChatGPT precludes definitive conclusions, several plausible factors may contribute to this behaviour. ChatGPT is trained on a large and diverse corpus extending beyond biomedical text, which may enhance robustness to stylistic and contextual variation in real-world clinical narratives. In addition, its optimisation for instruction-conditioned text generation and aligned interaction, further shaped by reinforcement learning from human feedback (RLHF), may support flexible interpretation of free-text clinical notes under distributional shift. Importantly, these same characteristics are not optimised for token-level classification, which likely contributes to ChatGPT’s weaker performance on structured benchmark tasks. These interpretations are necessarily speculative and should be understood as hypotheses rather than definitive explanations.

### Interpreting ChatGPT’s lower performance on public benchmarks

While ChatGPT performed comparatively well on the local EHR dataset, it consistently underperformed relative to domain-specific medical LLMs when trained and tested on public benchmark datasets for both medical concept extraction and medical question answering tasks. This pattern was observed across evaluation metrics, including lower F1 scores for concept extraction and lower BLEU and ROUGE-L scores for question answering.

A key factor underlying this result is the mismatch between ChatGPT’s instruction-conditioned generative optimisation and the token-level, lexically constrained evaluation frameworks used in benchmark datasets such as I2B2, N2C2, and emrQA. Discriminative models fine-tuned explicitly for these tasks are better aligned with strict boundary detection and exact lexical matching criteria, whereas models optimised for free-text generation may produce clinically reasonable responses that diverge from reference annotations.

### Practical implications and example use cases

The findings of this study have several practical implications for the development and deployment of LLM-based systems in clinical settings. First, our results indicate that off-the-shelf medical LLMs trained exclusively on public benchmark datasets should not be assumed to perform reliably when applied to new clinical environments without prior local evaluation. In practice, this suggests that healthcare institutions considering LLM-based tools for clinical note processing, such as automated extraction of diagnoses, medications, or adverse drug events, should incorporate local adaptation and validation as a prerequisite for deployment.

Second, the observed improvements from local fine-tuning highlight a feasible and resource-efficient pathway for adapting LLMs to specific healthcare contexts without requiring training from scratch. For example, healthcare institutions seeking to automate information extraction from discharge summaries, support clinical audit workflows, or enable medical question answering could fine-tune existing models using modest, expertly annotated subsets of local clinical notes. This approach is particularly relevant in resource-constrained settings, where access to large-scale computational infrastructure remains limited.

Third, the task-dependent performance differences observed across models underscore the importance of aligning model choice with intended clinical use. Consistent with our findings, discriminative models fine-tuned for token-level classification may be more appropriate for structured tasks such as concept extraction for registries, surveillance, or quality monitoring, whereas instruction-conditioned generative models such as ChatGPT may be better suited to applications requiring flexible interpretation of narrative text, such as summarisation or free-text question answering. Careful model selection and local validation are therefore essential to avoid inappropriate deployment based solely on benchmark performance.

Finally, the degradation in performance observed under external validation has important implications for patient safety and clinical governance. Errors in extracting or interpreting clinical information, such as missed adverse drug events or incorrect attribution of diagnoses, could propagate downstream into decision support systems, audits, or research analyses. Our findings therefore reinforce the need for governance frameworks that mandate local performance assessment, transparent reporting of model limitations, and ongoing monitoring when LLMs are introduced into clinical workflows.

### Challenges, limitations, and future work

There are several key challenges in making models reliable in practice. Real-world data such as EHR databases are not pre-designed for LLM applications. The manual labelling of data and re-structuring of clinical notes to conform to the required formatting of LLMs is time-consuming and demanding for busy clinician experts, preventing rapid development of suitably accurate, reliable, and unbiased models. Their production also requires close collaboration between clinicians, data scientists and IT specialists, which further complicates the generation of models. In addition, ethical concerns around the sharing of patient data necessitate privacy-preserving measures for these data sources.

An additional methodological consideration concerns the granularity of data used for local fine-tuning. In this study, models were fine-tuned using data drawn from a single national healthcare system, which provided sufficient scale and heterogeneity to support robust evaluation while remaining feasible within existing governance and privacy constraints. Although finer-grained adaptation, such as regional or institution-level fine-tuning, may further align models with local documentation practices and patient populations, such approaches are often constrained by fragmented data availability, limited sample sizes, and restrictive data-sharing policies. One potential pathway to address these challenges is the development of inclusive, nationally representative datasets that better capture regional variation, or the use of privacy-preserving techniques such as federated learning to enable model adaptation across institutions without direct data sharing. Future work should systematically evaluate whether such strategies improve performance without compromising generalisability, scalability, or ethical data governance.

Inevitably our study has some limitations. The findings reflect the performance of models on a random subset of clinical notes extracted from one EHR dataset in South Asia. While this EHR dataset has national coverage and is therefore representative of data from the general population of Pakistan in terms of socio-demographics and medical history, it represents a smaller-scale basis for LLM training. The key to successful health innovation lies in its reliability and perception as fit for use by the populations of interest and key stakeholders. Alongside increasing the scale of training data, future work should also focus on the challenges of creating medical LLMs trusted by doctors, nurses, and front-line staff, and how these users envision the future role of these models in patient care. The testing of these models in patient care will not only allow further analysis of their technical validity but also allow patients and clinicians to discuss the cultural and ethical acceptability of novel health-focused LLM technology in local health settings. This is an important first step to ensure that local stakeholders have agency and ownership in the development of transformative health technology, including LLMs, going forwards, in ways that ensure they are safe, fair and beneficial for all.

## Conclusions

Despite the elevated interest in LLMs, their assessment in real-world clinical settings is lacking. This study evaluates the reliability of LLMs for clinical note processing tasks relevant to clinical decision-making by assessing their performance on a local dataset from a hospital in Pakistan. Given that most medical datasets suffer from the digital divide, we tested and independently validated LLMs on a large local EHR database. The database was first labelled by a team of clinical experts from the setting in context through expert consensus to review to contest and redress algorithmic decisions.

We recognise that equitable use of health innovations extends well beyond considerations of technological bias; it necessitates consideration of clinical explainability, acceptability, trust and local ownership such as can be assessed through regular stakeholder engagement and interaction. This is an important avenue for future research to ensure clinical practitioners, decision-makers, and patients and carers have agency and ownership in the development and implementation of transformative health technology including LLMs going forward.

In conclusion, our findings highlight the underperformance of medical LLMs in a local Pakistani setting compared to those in Global North settings revealing the data-driven biases in existing publicly available medical LLMs, which are predominantly pre-trained on data from high-income settings. This work suggests that pre-trained LLMs can be adapted for parsing clinical notes in specific clinical contexts, but only through careful fine-tuning on locally curated datasets. It emphasizes the need for continued scrutiny of LLMs and suitable corrective measures, such as regular fine-tuning on local data under the supervision of local context-aware clinical experts.

## Supplementary Information

Below is the link to the electronic supplementary material.

Supplementary Material 1

Supplementary Material 2

## Data Availability

The datasets generated and/or analysed during the current study are available in the following public repositories: I2B2 2010 Challenge Dataset (medical concept extraction with treatment, test, and problem labels): available at [https://portal.dbmi.hms.harvard.edu/projects/n2c2-nlp/](https://portal.dbmi.hms.harvard.edu/projects/n2c2-nlp)N2C2 Dataset (2018 Track 2 - Adverse Drug Events and Medication Extraction): available at [https://portal.dbmi.hms.harvard.edu/projects/n2c2-nlp/](https://portal.dbmi.hms.harvard.edu/projects/n2c2-nlp)emrQA Dataset (clinical question answering): available at https://emrqa.github.io/Summary-level data from the local hospital (SKMCH&RC) used in the study are presented within the manuscript. Access to these data may be restricted due to institutional and ethical regulations.

## Abbreviations

LLMs: Large Language Models
EHR: Electronic Health Records
MCE: Medical Concept Extraction
MQA: Medical Question Answering
DS: Discharge Summaries
SOAP: Subjective, Objective, Assessment, and Plan
SKMCH&RC: Shaukat Khanum Memorial Cancer Hospital and Research Centre
BIO: Beginning-Inside-Outside
NER: Named Entity Recognition
I2B2: Informatics for Integrating Biology & the Bedside
N2C2: National NLP Clinical Challenges
