# Supplementary material for: Evaluating large language models for clinical note processing: local fine-tuning and internal-external validation using electronic health records from South Asia
Source: BMC Med Inform Decis Mak. 2026 Feb 25;26:75. doi: 10.1186/s12911-026-03366-8 (PMC12988631; doi:10.1186/s12911-026-03366-8)
Supplement: Supplementary file 2 — Supplementary Material 2 [file 12911_2026_3366_MOESM2_ESM.docx]

**Appendix 1**

In order to investigate the potential reasons of the bias presented in results (figure), the detailed comparison between the datasets are presented in this appendix.

1. **Concept Extraction Analysis**
   1. **Datasets Comparison:**

Table 1. Data sources of fine-tuning datasets

| **I2B2 2010 Dataset** | **N2C2 2018 Dataset** | **SKM Dataset** |
| --- | --- | --- |
| **Challenge Topic: Relations** | **Challenge Topic: Adverse Drug Events & Medication Extraction** |  |
| **Data description:** Partners Healthcare, Beth Israel Deaconess Medical Center, and the University of Pittsburgh Medical Center (UPMC) contributed discharge summaries to the 2010 i2b2/VA challenge. In addition, UPMC contributed progress reports. A total of 349 training reports, 477 test reports, and 877 unannotated reports were de-identified and released to challenge participants. | **Data description:** 505 discharge summaries drawn from the MIMIC-III (Medical Information Mart for Intensive Care-III) clinical care database, selected using a query that searched for an ADE in the International Classification of Diseases code description of each record. | **Inpatient Discharge Summary (DS) Notes: (50)**  DS notes provide a comprehensive summary of a patient's hospitalization, including diagnostic information; procedures performed, medications administered, and post-discharge instructions. These notes contain details such as patient demographics, admission and discharge dates, primary consultants, and a detailed description of the patient's condition and management during admission.  **SOAP Notes: (150)**  SOAP notes follow a structured approach and consist of four sections: Subjective, Objective, Assessment, and Plan.   1. Subjective: Includes patient symptoms, history, and information provided by the patient or caregiver. 2. Objective: Contains objective observations, laboratory results, and imaging data. 3. Assessment: Provides diagnoses, problem lists, and summaries of the patient's health status. 4. Plan: Includes detailed plans for treatment, medications, follow-up, and other relevant actions. |

It is worth mentioning that the SKM dataset encompasses a specific domain focused on cancer and COVID-19 patient records which is a subset of the broader clinical context covered by open datasets.

- 1. **Distribution of concepts in datasets**

In the following, we described the distribution of concepts and the structure of the texts in each dataset. Table 2 shows the distribution of concepts in I2b2 dataset and the corresponding lables in SKM.

Table 2. Distribution of the labels in I2B2 2010 and SKM datasets.

| **I2b2 2010 dataset (Problem, Treatment, Test)** | | | | | |
| --- | --- | --- | --- | --- | --- |
|  | | **Problem** | **Treatment** | **Test** | **O Labels** |
| I2b2 2010 | Total no. of labels | 151558 | 79878 | 76681 | 1005919 |
|  | Labels proportion | 11.53% | 6.08% | 5.84% | 76.55% |
| SKM | Total no. of labels | 11864 | 6599 | 2415 | 119963 |
|  | Labels proportion | 8.42% | 4.69% | 1.71% | 85.18% |

Table 2 highlights the higher percentages of labels associated with problem, treatment and test concepts, and the SKM dataset, where proportions of these concepts are comparatively lower. Particularly noteworthy comparing to the 76% of O labels in I2B2 dataset, 85% of the labels in the SKM dataset lie outside of the specified concepts.

Table 3. Distribution of the Labels in N2C2 2018 and SKM datasets

| **N2C2 2018 dataset** | | | | | | | | | | | | |
| --- | --- | --- | --- | --- | --- | --- | --- | --- | --- | --- | --- | --- |
|  | | **Duration** | **Frequency** | **Strength** | **Form** | **Route** | **Dosage** | **Reason** | **ADE** | **Drug** | **O Labels** |  |
| N2c2 2018 | Total no. of labels | 2020 | 24320 | 16590 | 14220 | 7566 | 16806 | 9264 | 2226 | 26374 | 1052134 |  |
|  | Labels Proportion | 0.17% | 2.08% | 1.42% | 1.21% | 0.65% | 1.43% | 0.79% | 0.19% | 2.25% | 89.81% |  |
| SKM | Total no. of labels | 4316 | 3669 | 5084 | 2478 | 1888 | 1397 | 152 | 13 | 4938 | 119669 |  |
|  | Labels Proportion | 3.01% | 2.55% | 3.54% | 1.73% | 1.31% | 0.97% | 0.11% | 0.01% | 3.44% | 83.33% |  |

Table 3 explains the contrast between the N2C2 2018 and the SKM datasets, particularly regarding medication-related labels. For instance, while 'Duration' accounts for only 0.17% of labels in the N2C2 2018 dataset, it constitutes 3.01% of labels in the SKM dataset. Additionally, 'ADE' accounts for 0.19% of labels in the N2C2 2018 dataset compared to just 0.01% in the SKM dataset. In this case, in contrast to the Table 2, the proportion of O labels in N2C2 is higher than SKM dataset.

- 1. **Structure of notes in each dataset**
     1. **SKM note structure**

In the SKM datasets, medication details in the discharge summaries and SOAP notes are presented as follows:

1. **Discharge summaries**

Medication information is organized into different sections using the following formats:

1. “Medication to take at home”

(Drug Form Strength Frequency Duration Route)

1. “Medication administered during the admission”

( Drug Form Strength Frequency Duration)


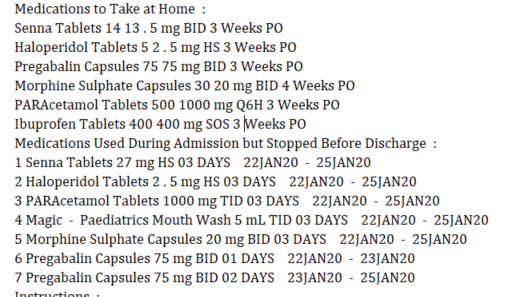

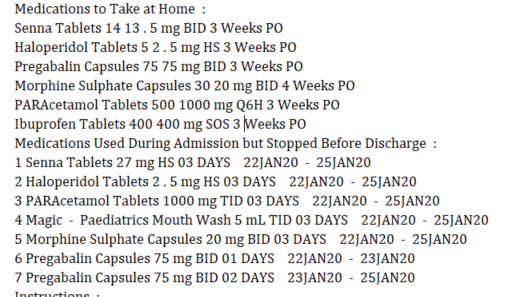


Figure 1. SKM discharge summary sample

1. **SOAP Notes**

Following formats are used to show the medication information into different sections.

1. “Treatment Options and recommendations”

(Drug Strength Route Frequency)

1. “Drugs prescribed today”

Drug Form Strength Dosage Form Frequency Duration


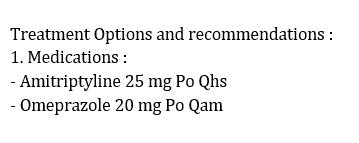

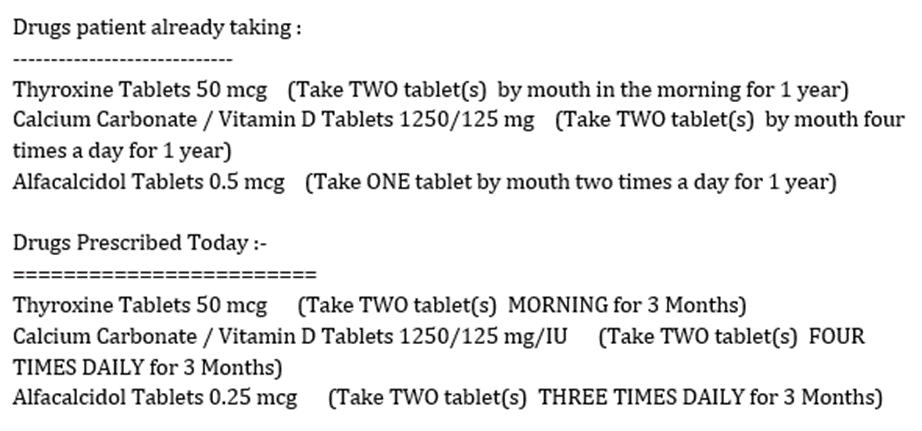


Figure 2. SKM SOAP note sample

- - 1. **Open datasets note structure**

For the n2c2 dataset, the medication formats in these notes is not uniform, as they vary depending on the context. Typically, medications are listed under headings "Medications on Admission" and "Discharge Medications." Additionally special instructions are provided for specific medications, such as warnings not to drive after taking certain medications or the reasons for prescribing them.

Following are some example formats that are used for medication information in different sections.

**Example-1**

1. Medications on Admission

- **(Drug Strength Form Frequency)** Nadolol
- **(Drug)** Lactulose, Mvi

1. Discharge Medications:

- **(Drug Strength Form Route Frequency )** 1-5, 7
- **(Drug Strength Form Frequency Route Duration)** 6 Ciprofloxacin 500 mg


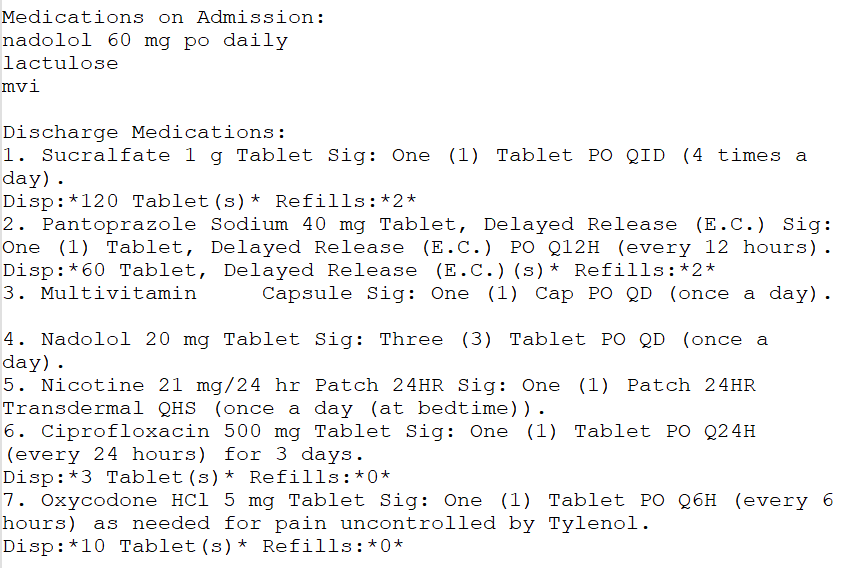


Figure 3. Sample medication note of N2C2 dataset

**Example-2**

1. Medications on Admission
   - **Drug Strength Frequency**
   - 1. Cyclosporine 50 mg twice daily
   - **Drug Strength Frequency Strength Frequency Strength Frequency**
   - 3. Prednisone 20 mg Daily, 15 mg qam, mg qpm
2. Discharge Medications:
   - **Drug Strength Form Dosage Form Duration:**
   - 19. Prednisone 20 mg Tablet Sig: 0.5 Tablet PO QPM for 5 days.
   - 20. Prednisone 20 mg Tablet Sig: 1.5 Tablets PO QAM for 5 days.
   - **Drug Strength Form Dosage Form Route Frequency:**
   - 9. Pantoprazole 40 mg Tablet, Delayed Release (E.C.) Sig: One (1) Tablet, Delayed Release (E.C.) PO Q12H (every 12 hours).


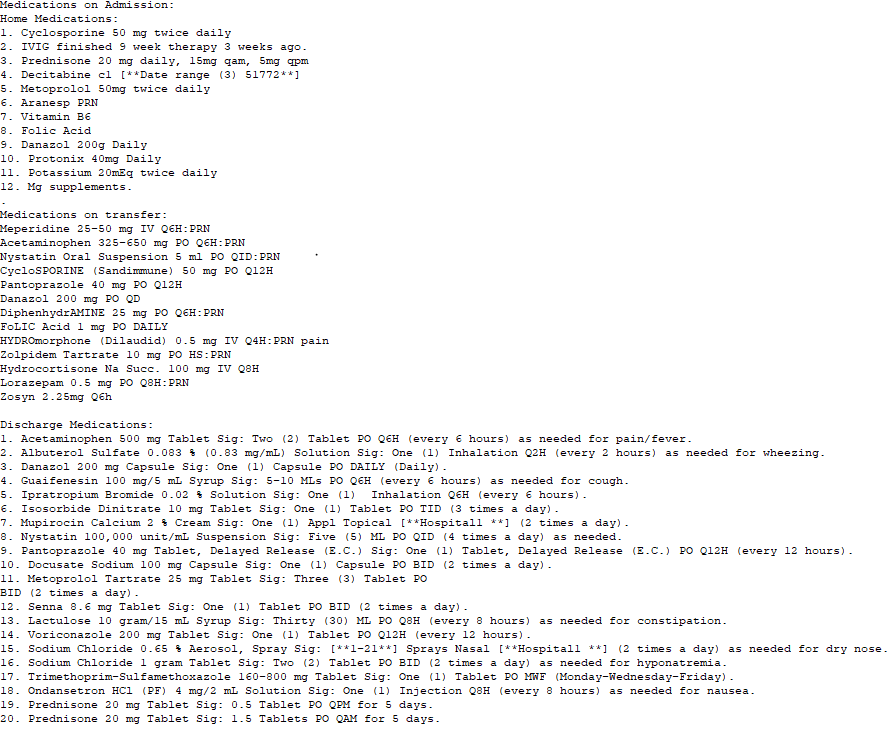


Figure 4. Sample medication note of N2C2 dataset

1. **Question Answering Analysis**
   1. **Difference Between emrQA and SKM Datasets**

Below is a comparison between SKM and emrQA discharge summaries. Both summaries exhibit a similar structure. Through our random analysis of the emrQA data, we observed various formats, with most featuring multiple medication groups tailored to different specialties. We have included the most prevalent format below for reference.

Table 4. Comparison of the note structures

| **SKM** | **emrQA** |
| --- | --- |
| **Patient Information:**  Admitting Consultant, Admission Date, Discharge Date | **Admission Details:**  Admission Date, Discharge Date, Discharge Summary Status |
| **Reason for Admission:**  Admission Reason | **History of Present Illness:**  Patient Information, Weight History, Mobility and Symptoms |
| **Background Diagnosis:**  Background Diagnosis | **Past Medical History:**  Heart Murmur, Duodenal Ulcer, Diabetes Mellitus, Laparotomy |
| **Diagnostic Tests Performed:** Procedure details performed during admission | **Procedure:** Procedure details performed during admission |
| Diagnosis details are mentioned in the background diagnosis section | **Diagnosis:** Diagnosis details |
| **Significant Findings on Admission:**  Findings on Admission | **Physical Exam on Admission:**  Vital Signs  Height and Weight  General Exam |
| **Medications Administered During Admission but Stopped Before Discharge (with Dates):**  List of Medications with Dates | **Medications:** List of Medications of administered during admission. |
| **Hospital Course and Management During Admission:**  Procedures  Condition at Discharge | **Hospital Course:**  Admission Reason, Panniculectomy, Postoperative Progress  Discharge Details |
| **Medications to Take at Home:**  List of Medications | **Discharge Medications:**  List of Medications |
| Physician information mentioned in the patient information heading | **Dictated By:**  Attending Physician Information |
| **Discharge Instructions:**  Follow-up Instructions  Contact Information in an Emergency | **Follow up Appointments** |
| **Significant Tests/Problems to Address on Follow-up:**  Follow-up Tests/Problems |  |

- 1. **Comparison of medication information in datasets**

The following table presents some differences in the medication text available in two datasets.

Table 5. Medication text comparison in datasets

| **SKM** | **emrQA** |
| --- | --- |
| In the SKM dataset, like EMRQA, medications are divided into 'Before Discharge' and 'At Home' categories. SKM often features repeated medications with different dosages and frequencies, distinguished by 'Before Discharge' and 'At Home' labels in questions. These details are absent in EMRQA, highlighting a key difference between the two datasets. | In some cases, the same medications may be repeated in the summaries, with consistent details regarding dosage and frequency. In such scenarios, models usually prioritize the initial occurrence. |
| In the emrQA dataset, there is no information regarding the dates of medication prescriptions, whereas in the SKM dataset, each medication is accompanied by prescribed dates. | In a small subset of cases within the emrQA dataset, when multiple occurrences of the same medication with different dosages and frequencies are present, identical questions are generated for each instance. However, these questions lack distinguishing elements to differentiate between the various cases. |
|  | In the emrQA dataset, additional information regarding the reason for prescribed medication is available, whereas such information does not exist within the SKM dataset. |
